# A syndemic born of war: Combining intersectionality and structural violence to explore the biosocial interactions of neglected tropical diseases, disability and mental distress in Liberia

**DOI:** 10.1371/journal.pgph.0000551

**Published:** 2022-06-29

**Authors:** Laura Dean, Sally Theobald, Gartee Nallo, Anthony Bettee, Karsor Kollie, Rachel Tolhurst

**Affiliations:** 1 Department of International Public Health, Liverpool School of Tropical Medicine, Liverpool, United Kingdom; 2 University of Liberia Pacific Institute for Research and Evaluation, Monrovia, Monsterrado, Liberia; 3 Neglected Tropical Disease Programme, Ministry of Health, Government of Liberia, Monrovia, Monsterrado, Liberia; Emory University, UNITED STATES

## Abstract

The intersections between NTDs, disability, and mental ill-health are increasingly recognised globally. Chronic morbidity resultant from many NTDs, particularly those affecting the skin—including lymphatic filariasis (LF), leprosy, Buruli ulcer (BU) and onchocerciasis—is well known and largely documented from a medicalised perspective. However less is known about the complex biosocial interaction shaping interconnected morbidities. We apply syndemic theory to explain the biosocial relationship between NTDs and mental distress in the context of structural violence in Liberia. By advancing syndemic theory to include intersectional thought, it is apparent that structural violence becomes embodied in different ways through interacting multi-level (macro, meso and micro) processes. Through the use of in-depth qualitative methods, we explore the syndemic interaction of NTDs and mental distress from the vantage point of the most vulnerable and suggest that: 1) the post-conflict environment in Liberia predisposes people to the chronic effects of NTDs as well as other ‘generalised stressors’ as a consequence of ongoing structural violence; 2) people affected by NTDs are additionally exposed to stigma and discrimination that cause additional stressors and synergistically produce negative health outcomes in relation to NTDs and mental distress; and 3) the impact and experience of consequential syndemic suffering is shaped by intersecting axes of inequity such as gender and generation which are themselves created by unequal power distribution across multiple systems levels. Bringing together health systems discourse, which is focused on service integration and centred around disease control, with syndemic discourse that considers the biosocial context of disease interaction offers new approaches. We suggest that taking a syndemic-informed approach to care in the development of people-centred health systems is key to alleviating the burden of syndemic suffering associated with NTDs and mental distress currently experienced by vulnerable populations in resource-limited settings.

## Introduction

### What are syndemics?

Syndemics can be described as ‘*synergistically related’ epidemics that cluster around harmful conditions’*. Syndemics occur where social and structural inequalities create increased physical and behavioural vulnerabilities to ill-health, resulting in the clustering of two or more diseases within a population [1,2]. Often, within syndemic discourse, multiple conditions or health problems are found to interact biologically resulting in worsening health outcomes [2]. Through blending multiple theories from the social and medical sciences, syndemic theory supports the development of understandings of disease experience beyond biological and epidemiological clustering to consider how social, political and economic forces shape the relationships between diseases and illness experiences to perpetuate health disparities and poor social condition [1–3]. However, robust empirical analysis of syndemic relationships has been described as lacking [3].

### Neglected tropical diseases, disability and mental ill-health

Neglected Tropical Diseases (NTDs) are a group of acute and chronic infections that frequently occur as a result of poverty, poor environmental conditions and social disadvantage [4,5]. The intersections between NTDs, disability, and mental ill-health are increasingly recognised globally [6,7]. Chronic morbidity resultant from many NTDs, particularly those affecting the skin—including lymphatic filariasis (LF), leprosy, Buruli ulcer (BU) and onchocerciasis—is well known and largely documented from a medicalised perspective [6,8]. Additionally, studies over the last five years have revealed significant co-morbidity between NTDs and depressive disorders [6,7]. Highlighting epidemiological associations between NTDs, disability, and mental disorders is critically important; however, it is now widely recognised that understanding the biosocial connections between diseases is essential in responding to disease interactions and their impact on the health and wellbeing of affected populations [1,6,9,10]. The current focus on physical and mental co-morbidities associated with NTDs is generally framed within a bio-medical perspective, with limited consideration of the bio-social context [11]. Thus, an evidence gap remains in understanding the social and structural processes that shape disease interactions between NTDs, disability and mental ill-health.

### Syndemic theory and neglected tropical diseases: An opportunity to advance theory and practice

The application of syndemic theory to understanding the experiences of people affected by NTDs, presents a unique opportunity to interrogate the biosocial connections between NTDs and mental distress; enhancing our theoretical understandings of existing epidemiological associations whilst also providing knowledge to guide the most appropriate health systems responses. We have used an in-depth qualitative methodology, to explore lived experience of NTDs from the viewpoint of affected populations to develop explanations and understandings of how forms of power at the macro (national/political-economy), meso (institutions and social processes) and micro (individual and household) level interact to shape specific outcomes in relation to health and wellbeing [3,9]. In-depth qualitative data can thus provide the individual and community level foundations that support the explanation of macro (national)-level observations established through epidemiological studies [3].

Despite epidemiological evidence of co-morbidities between mental distress and NTDs, to the best of our knowledge, the biological interactions between NTDs considered in this manuscript (onchocerciasis, leprosy, Buruli ulcer, lymphoedema, hydrocele) and mental distress have never been considered. As part of our exploration, we have not explored biological interactions between disease conditions; rather we seek to shed light on the social and structural mechanisms through which mental distress and NTDs synergistically interact to create exacerbations of health disadvantage. In advancing the syndemic argument in relation to NTDs and mental distress, further study of biological interactions would also be required [12]. However, our application of anthropological fieldwork into the study of syndemic suffering (the lived experience of the syndemic clustering of disease) [13] supports the advancement of empirical analysis of this syndemic relationship, whilst providing learning for other syndemic studies [3].

### A focus on equity: Structural violence and intersectionality

Structural violence, as a critical underpinning of syndemic theory [1,3], encourages detailed consideration of how unequal global and local political-economies and social organisation inflict embodied harm on people [14]. These macro-level determinants do not, however, impact all individuals in the same way or operate in isolation, rather, they intersect with individual identities (which are themselves constituted by intersections between sex, age, economic status etc.) to shape nuanced and shifting experiences and outcomes for individuals within populations [15–17].

In considering syndemic interactions [1] between NTDs, disability and mental ill-health, it is therefore important to consider the operation and intersection of power relations at multiple levels, from the macro to micro. Structural violence, when used as an analytical framework in isolation, has been critiqued for failing to consider how interactions between varying forms of violence (physical, economic, political and social) interact and are mediated by characteristics at the micro or individual level to create specific and nuanced experiences of health and social inequalities [18]. Intersectional theory supports such considerations as it enables exploration of how the multiple social locations of individuals intertwine to shape health inequities that result from a web of mutually reinforcing and intersecting power relations creating a ‘specific matrix of domination’ [9,15–17,19–24]. Intersectionality provides us with the opportunity for a cumulative exploration of individuals micro-positioning within both macro structural and social processes [20], supporting the understanding of the complexity of people’s lived realities of relationships and responses to health and illness [25]. Thus, when applied together as analytical tools, intersectional theory and structural violence have the potential to advance the empirical study of syndemics, supporting explorations of how syndemic interactions are underpinned by mutually reinforcing systems of both structural and social oppression to perpetuate individual suffering in specific contexts [9,20,26,27].

### The context of Liberia

Liberia has a deeply complex political and social history as a result of a unique post-slavery, post- colonial experience. Deeply rooted disunity between ‘settlers or colonists’ (freed African American slaves who had declared Liberia an independent nation in 1847 from the American Colonisation society) and indigenous peoples are frequently cited as the roots of protracted unrest and fragility [28,29]. These fractures resulted in 25 years of gross economic mis-management (GDP fell by 90% in less than two decades) and a 14-year civil war [28–30]. 2003 marked the end of two periods of conflict, both of which devastated the country’s health and social infrastructure, contributing to widespread extreme poverty, an absent education system, and a lack of institutional capacities [29–32]. For many Liberians, this nexus of factors shapes poor health outcomes and has created a reliance on alternative forms of health care such as traditional medicine [32]. Most recently the Ebola outbreak triggered the almost entire collapse of the Liberian health system [33], and the COVID-19 pandemic has placed additional strain on an already over-stretched health system.

### Our contribution

In this paper, we use in-depth qualitative methodology to explore the case for a syndemic relationship between NTDs and mental distress in the context of structural violence in Liberia. We use the term mental distress to refer to descriptions of stress, anxiety, depression and suicidal thoughts within participant accounts. We argue that a key pathway through which NTDs and mental distress are linked is as a result of NTD-related chronic morbidity (e.g. lymphoedema) and disability (as a result of associated stigma and social exclusion). However, not all persons affected by NTDs experience chronic morbidity and or disability. We therefore use the term NTDs within this paper to encompass all forms of disease experience, including chronic morbidity and disability.

By drawing on Meyer’s (2003) ‘minority stress model’ [34] and Medenhall’s (2017) [2] model of syndemic approaches to health, we suggest that: 1) the post-conflict environment in Liberia predisposes people to the chronic effects of NTDs as well as other ‘generalised stressors’ (e.g. poverty) as a consequence of ongoing structural violence; 2) people affected by NTDs (as a minority status) are additionally exposed to both ‘internal and external minority stressors’ (largely driven by stigma and discrimination) that synergistically produce negative health outcomes in relation to NTDs and mental distress; and 3) the impact and experience of both generalised and minority stressors and consequently syndemic suffering is shaped by intersecting axes of inequity such as gender and generation which are themselves created by unequal power distribution across multiple systems levels. Finally, by drawing on narrative methods and intersectional analysis we explore the nuanced experiences and realities of syndemic suffering from the vantage point of affected populations, and consider how such approaches aid in supporting the design of multi-level person-centred responses to syndemics in LMICs.

## Methods

**Fig 1:**
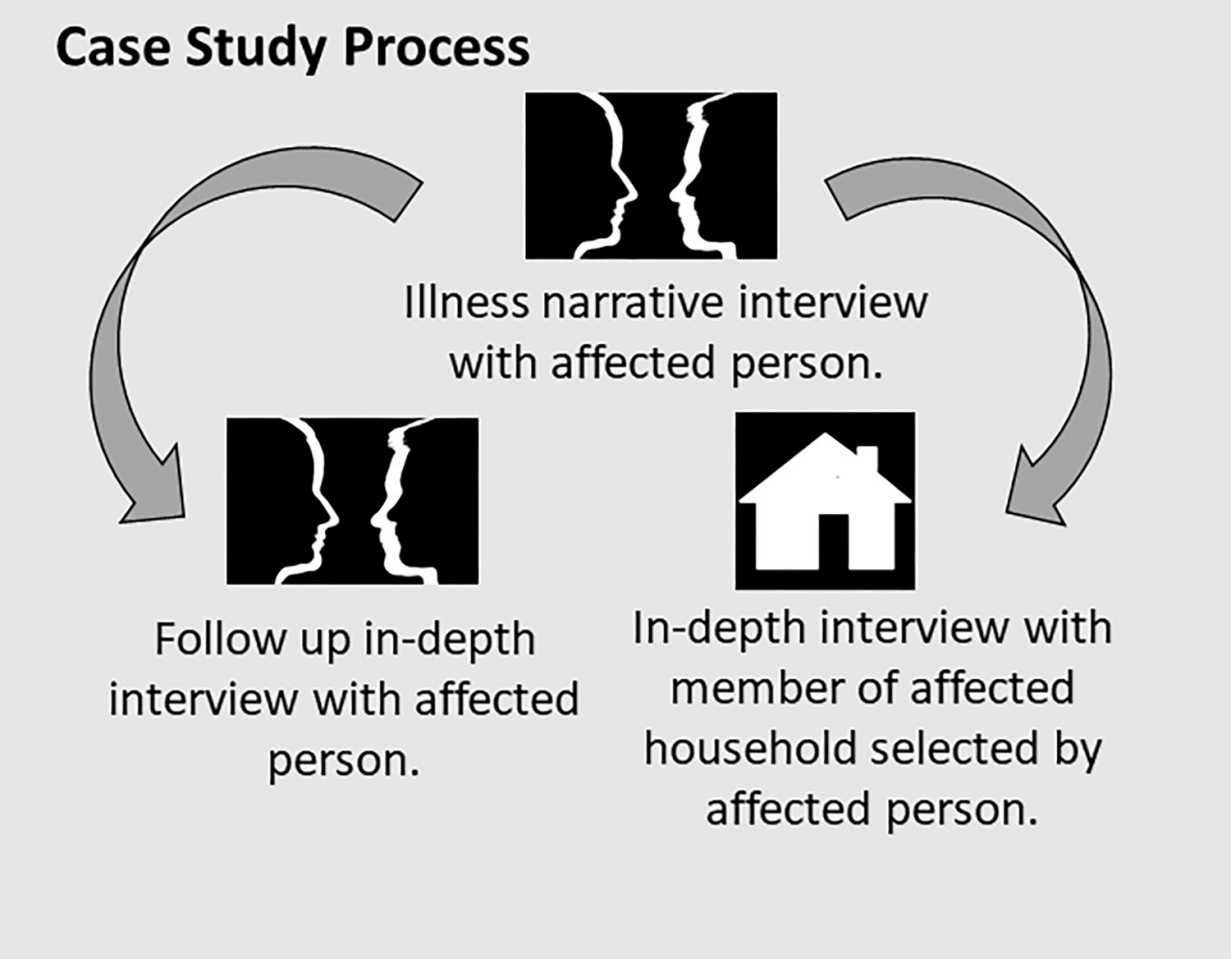
Case study process.

We used a case study method to explore illness experience from the perspective of individuals living with clinical manifestations of one or more of the identified NTDs (Fig 1). In addition to these narrative and follow up in-depth interviews, to complete case study sets, semi-structured interviews were completed with household members as within NTD literature there is evidence to suggest that suffering in relation to NTDs (specifically leprosy) has impact at the household level [7,35]. Case studies were complemented with the use of key informant interviews to ensure further consideration of the broader socio-political context in relation to NTDs and mental distress in Liberia.

Situated within the naturalistic paradigm of qualitative research enquiry [36,37], narratives seek to redress the dominance of biomedicine within research on health inequities by exploring the impact of multiple structural and social oppressions on health and wellbeing from the vantage point of the most marginalised [19,38]. By using everyday life as an entry point, narrative approaches resituate the human as a storyteller best placed to recount their own reality of suffering and resilience [27,38]. Additionally, narratives can support individuals to make sense of their illness experience [27] in relation to varying and interconnected social categories and identities [39]. Thus, narratives are a useful tool both in advancing syndemic theory [27] and in intersectional research [38].

### Study setting and disease focus

We collected data in three purposively selected counties: Maryland, Nimba and Bong. Selection criteria included: 1) all diseases are endemic; 2) geographical and socio-cultural diversity; 3) differing levels of health systems infrastructure; and 4) pilot sites for the roll out of the Liberian Ministry of Health’s strategy for the integrated case management of NTDs. Within each county, we further purposively selected study district(s) (one in Maryland, one in Bong and three in Nimba) based on NTD-associated morbidity identified from health records. Maximum variation across all study districts was aimed for in geography (rural/peri-urban, border/non-border) and socio-cultural context (ethnicity and language).

Liberia’s integrated case management plan for NTDs focuses on Leprosy, BU, Yaws and the clinical manifestations of LF (lymphoedema and hydrocele) [40]. In this study, we focused on Leprosy, BU, lymphoedema and hydrocele. Onchocerciasis was also included due to the large numbers of persons affected by chronic morbidity as a result of high disease endemicity in Liberia. Yaws was excluded because when the study commenced cases of yaws were not yet confirmed in Liberia.

### Participants and sampling procedure

#### Illness narrative case studies

We generated a sampling frame of all potential participants from health systems records and tacit knowledge of health workers. We then purposively selected participants to ensure maximum variation, considering age, gender, and experienced morbidity as core sampling criteria. However, as case studies were generated we ensured that further diversity in the biological and social conditions of participants was achieved; this was in response to reflection participant descriptions of their lives both pre and post illness and researcher observation of the broader environment where participants resided. Table 1 provides an overview of the case studies completed and S1 Text gives a summary of each case study. To identify household members to include in case studies affected persons were asked to identify someone of significance in their daily lives that we could also talk too. Case study data collection continued until data inductive thematic saturation was reached; that is when core analytical themes became repetitive and emergence of new themes or ideas infrequent [41].

**Table 1:** Summary of case studies completed.

	Age	LF (Lymphoedema/Hydrocele)	BU	Onchocerciasis	Leprosy	Total	
**Men**	**18–24**				2	**2**	**14**
	**25–49**	2	2	1	2	**7**	
	**Over 49**	1		2	2	**5**	
**Women**	**18–24**		2			**2**	**13**
	**25–49**	3	2	1	1	**7**	
	**Over 49**			1	3	**4**	
**Total**		**6**	**6**	**5**	**10**	**27**	

#### Key informant interviews

We conducted thirteen individual and one paired semi-structured interview with key informants at the national and county level. Table 2 provides an overview of issues explored, key informant type (purposively selected based on their job role) and their health systems function. A priori thematic saturation was achieved within key informant data as the purpose for key informant inclusion was to ensure clearer understanding of the socio-political context in relation to NTDs, disability and mental ill-health [41].

**Table 2:** Key informant characteristics and issues explored.

Key Informant Type	Role in Health System	Number Included	Issues Explored
NGOs or Donor Representative	Provide programme funds and technical support to implementation through the same organisation.	2	•Disability •Mental Health •NTDs •Generation, content and implementation of Integrated Case Management Plan •Programme strengths and challenges
National Ministry of Health Staff (NTD Programme)	Oversee NTD policy development and programme delivery.	4	
National Ministry of Health Staff (Rehabilitation Department)	Oversea rehabilitation policy development and service delivery.	1	•Disability •Mental Health •NTDs •Vignettes developed from narrative case studies presented to explore participant reactions to experiences and develop holistic solutions.
National Ministry of Health Staff (Mental Health Department)	Oversea Mental Health policy development and service delivery.	1	
Disabled People’s Organisations	Civil society organisation aimed at promoting collective action amongst disabled persons and rights advocacy.	2	
Ministry of Health County Health Team (NTDs and Mental Health Focal Points)	Support the implementation of NTD programme and mental health service delivery at county level.	1	

#### Data collection process

Data collection took place between January 2017 and June 2018.

#### Illness narrative case studies

Initial narrative interviews with affected persons took a highly unstructured approach to questioning to allow for subjective reflection [37]. The initial interview strategy drew on life history approaches to understand participants’ background [42]. This involved giving participants the space to speak freely about key events in their lives [27], before encouraging them to focus on illness experience linked to NTDs [43]. We guided participants to consider areas of participation, as guided by the international classification of functioning [44]. Participation domains included: learning and applying knowledge; general tasks and demand (such as daily routine); communication; mobility; self-care; domestic life; interpersonal interactions and relationships; major life areas (such as education and employment); and community and social life. Although these areas were identified as important if the natural flow of the discussion did not allow for them to be raised, they were explored during the follow up interviews.

The purpose of household member interviews was to gain additional understanding of the experience of affected persons and household impacts within the broader social context. Therefore, a semi-structured approach was used to guide participants through multiple and diverse topic areas within one interaction [45]. Interviews explored: knowledge and perceptions of disease and/or disability (including mental ill-health); impact of the disease on the individual and household (including relationships, daily routine, economics, community interactions); and possible support interventions.

### Ethical considerations

Written or witnessed verbal informed consent was obtained from all participants. Ethical considerations are discussed in detail in *Dean et al* (2019) [46]. Supporting the wellbeing of participants was prioritised throughout, which involved ensuring appropriate medical and psycho-social support was provided where required. Names used in presenting the data are pseudonyms. Ethical approval was granted from the Liverpool School of Tropical Medicine (16–070) and by the University of Liberia, Pacific Institute for Research and Evaluation Institutional Review Board (17-02-024).

### Data analysis

The primary focus throughout analysis was to privilege the voice of affected persons and other members within the household. Analysis drew on the use of thematic analysis, as well as analytical steps that allowed for the holistic consideration of case studies, since both narrative and intersectional analysis require holistic consideration of stories in relation to the broader socio-political context [47]. Analysis was ongoing throughout the data collection period, including: critical reflection of emergent issues and themes during the first interaction with participants; re-listening to initial interviews; and design of questions to explore key themes and emergent issues in subsequent interviews and additional case studies. All transcripts (which were transcribed verbatim into English) within a case study were used to develop a summary of the case study. Key threads and themes within each narrative were identified and considered in relation to the broader temporal and social context. From these summaries a very broad coding framework was developed and used to explore links and patterns across narratives [37]. We applied this coding framework to the data (using NVIVO 11 software), and charted data into broader themes to develop an explanatory account [45]. Key informant interviews were also analysed thematically and charts from the different data sources brought together at explanation stage.

**Fig 2:**
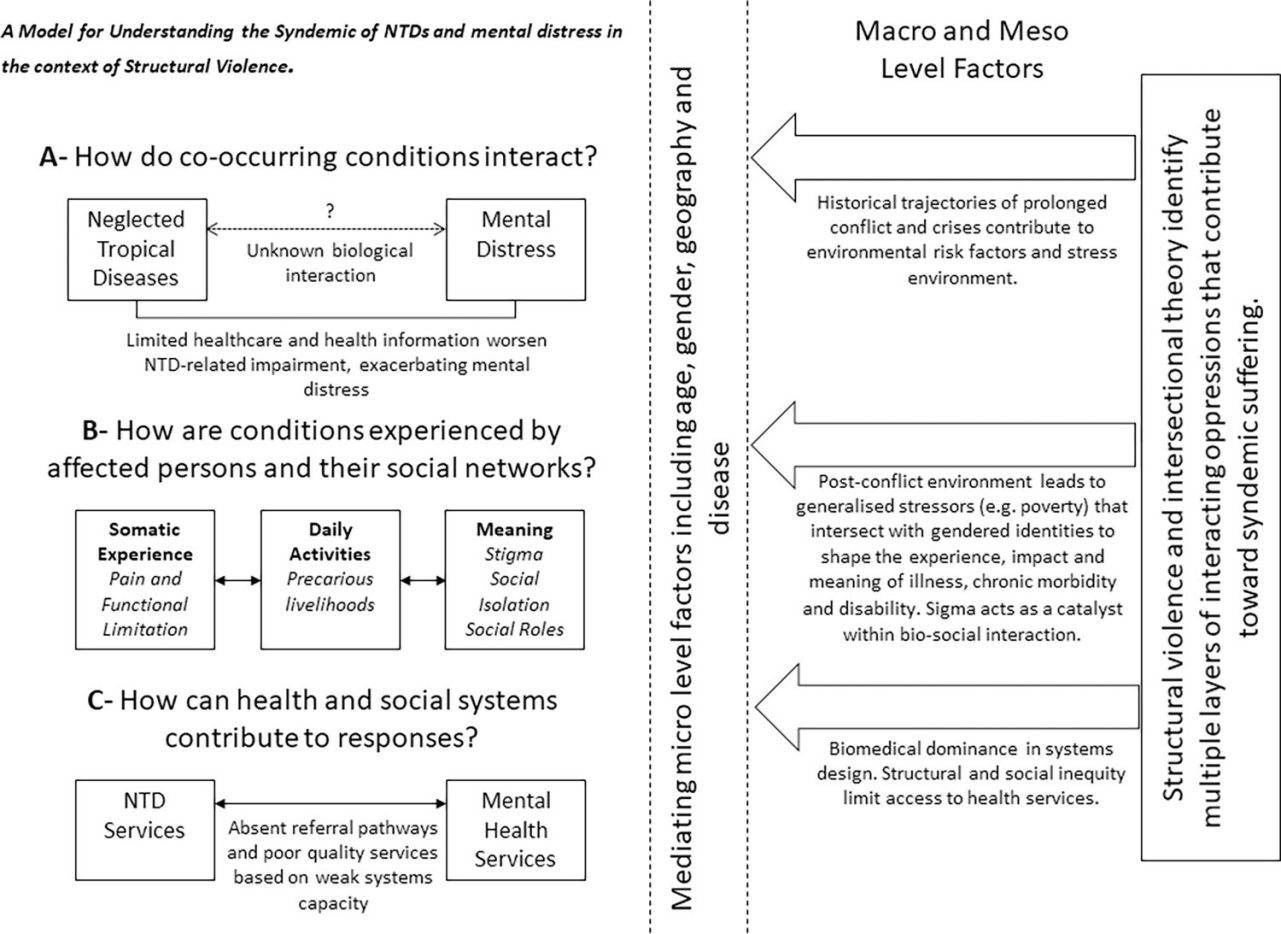
A model for understanding the syndemic of NTDs and mental distress in the context of structural violence.

We used theories of structural violence and intersectionality to inform analysis and support triangulation across methods to understand how the syndemic of NTDs and mental distress is shaped by intersecting factors at the macro, meso and micro level [17]. We draw on both intra- and inter-categorical intersectional analysis as we explore experiences of specific sub-groups within the larger group of people affected by NTDs whilst also exploring the complexity and relations between multiple categories of inequality [48]. In generating explanatory accounts of data, Mendenhall’s (2017) model for syndemic approaches to health was applied and adapted as presented in Fig 2 [2]. This enabled discussions around meaning and linkages within our data as well as group reflections on appropriate health systems responses.

## Results

Our findings present our adaptation of Mendenhall *et al*’s (2017) ‘Model for Syndemic Approaches to Health’ [2] (Fig 2) to present evidence of a syndemic relationship between NTDs and mental distress in the context of structural violence in Liberia. Stigma is described as a key structural mechanism through which this syndemic interaction is promoted [49]. Intersectional theory becomes critical in shaping understandings of how historic forces of power and oppression (that underlie structural violence) interconnect to contribute toward ‘syndemic vulnerability’. Specifically, intersectionality allows for consideration of how individual positionalities within a web of intersecting inequities shape how the stress environment becomes embodied in individual experiences of syndemic suffering (Fig 2, Section B). Our analysis revealed multiple multi-level pathways through which structural violence shapes vulnerability to NTD infection, chronic morbidity/disability, and mental distress in ways that reinforce each other. We discuss these in turn in the following sections as they are represented in Fig 2.

**Fig 3:**
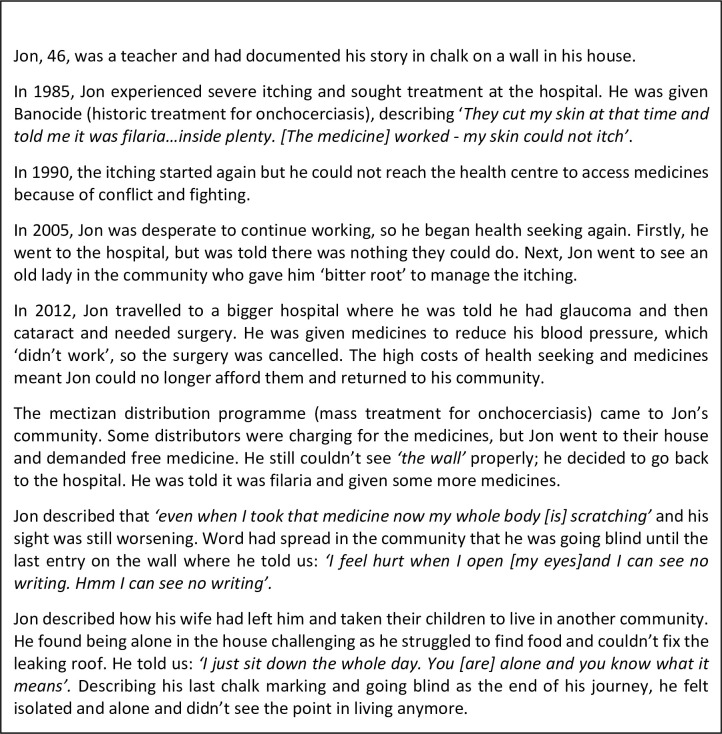
Jon’s story.

**Fig 4:**
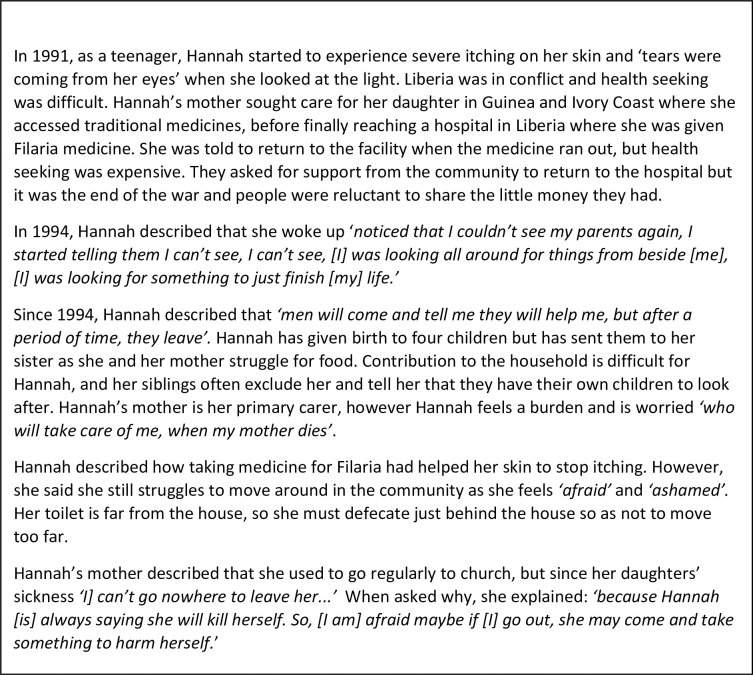
Hannah’s story.

**Fig 5:**
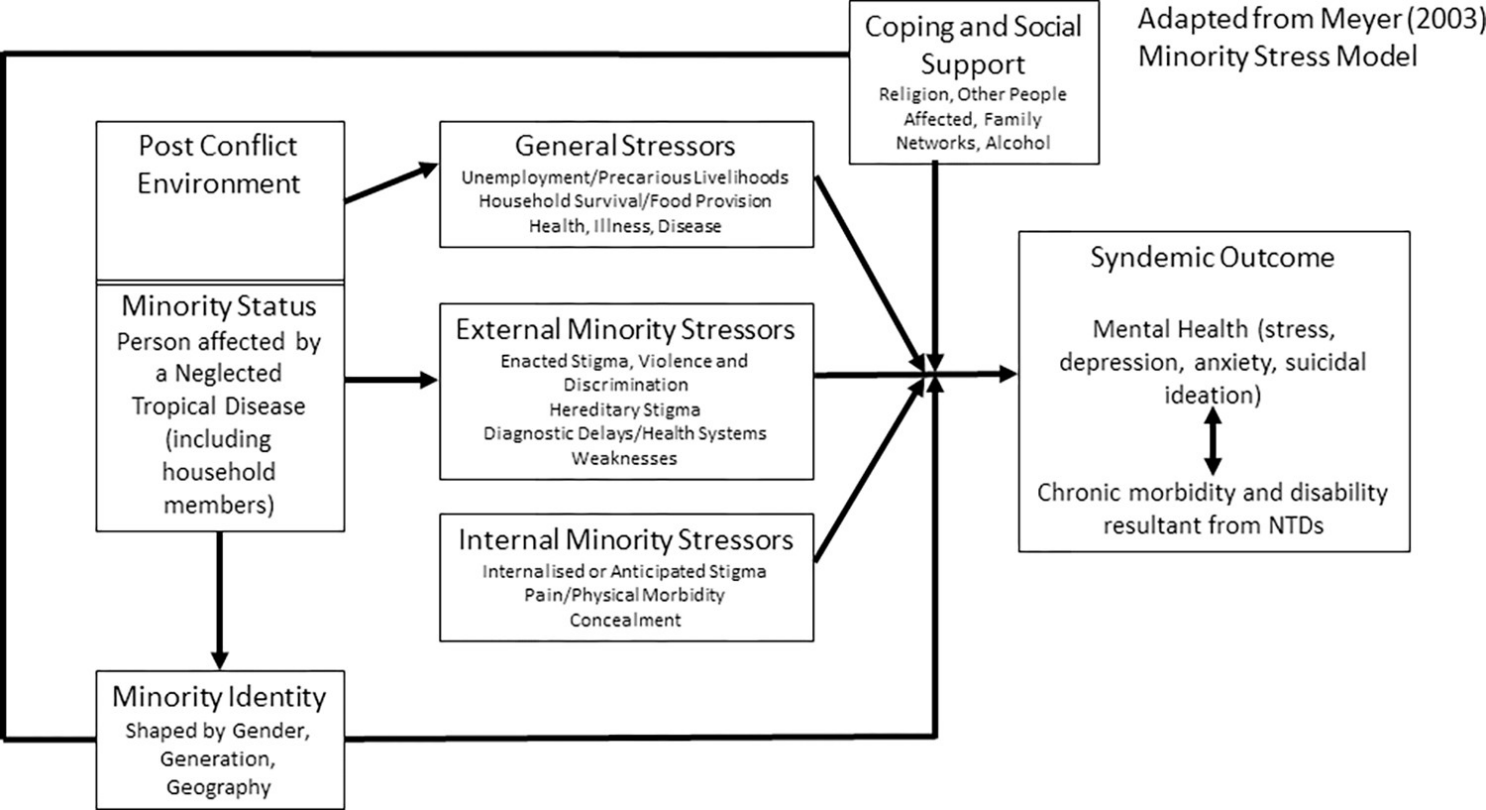
Adapting Meyer’s (2003) minority stress model [34] to emphasise the catalytic role of stigma in shaping syndemic outcomes for people affected by NTDs.

Before exploring the syndemic, we first introduce two stories that emphasise the suffering endured in relation to NTDs and mental distress in Liberia. Both Jon’s Story (Fig 3) and Hannah’s Story (Fig 4), characterise the lived reality of syndemic suffering by highlighting how larger structural socio-political processes such as protracted conflict and poverty (macro) and resultant weak health and social systems (meso) have created **general stressors** (Fig 5 factors that may cause stress to all persons in Liberia) that particularly affect people living with NTDs, (as a minority status), creating a risk environment for exacerbated physical morbidity and mental distress. **Internal and external minority stressors** (Fig 5 factors that may cause stress that are unique to persons affected by NTDs) related to stigma and discrimination (meso-micro) as a result of NTDs, catalyse a synergistic interaction between NTDs and mental distress that are further shaped by household and individual (micro) level characteristics that **shape minority identity** (Fig 5), such as gender and generation, to create nuanced experiences of syndemic suffering.

### How do the social-psychological processes of co-occurring conditions interact?

There are no studies in Liberia, and few internationally, that have interrogated the epidemiological overlap between NTDs and mental distress [7,50,51]. Where epidemiological overlaps have been considered, rates of mental distress amongst people affected by NTDs have been shown to be higher than in the general population [50,52,53]. A study in Ethiopia found that people with lymphoedema (as a result of podoconiosis) scored 1.43 points higher [95% CI:0.52 to 2.34] on the Kessler psychological distress scale than healthy neighbourhood controls [50]. High rates of depressive disorders were also found amongst people affected by filariasis in Togo (70%) [54] and India (97%) [55], and estimated between 12.5% and 76% among various leprosy affected populations [52,56–59]. The reverse correlation has not been considered, however it is hypothesised as based on overlapping risk factors such as poverty, social exclusion, and poor living conditions [7,50].

Data on mental health and NTDs in Liberia is lacking due to a lack of research and weak surveillance systems [40,60]. Available data shows that following the conflict in Liberia there is a high burden of mental ill-health with 40% of the population self-reporting symptoms consistent with major depression, and 44% of the population describing symptoms common with or experience of post-traumatic stress disorder(PTSD) [61]. PTSD symptoms have also been documented to have a longer term impact than is typical due to the extent of violence experienced during Liberia’s conflict and the protracted experience of material deprivation [62]. Simultaneously, the ongoing context of poverty, poor environmental conditions (including inadequate water and sanitation infrastructure), low levels of literacy and weak health service provisions means that many Liberians are vulnerable to NTDs and their associated chronic morbidities [4,63]. Although exact burdens are unknown, due to a lack of case reporting and identification, the Liberian Ministry of Health, has identified that NTDs, particularly those manifesting in the skin, present a substantial disease burden [40]. Following the first year of integrated active case detection for the NTDs of focus in this study, diagnosis and detection rates rose dramatically; for example, the number of health system reported cases rose from 44 to 327 (439% increase) in Maryland county alone [64]. Patients accessing treatment for the first time were identified as presenting in the latter stages of disease. For example, at the point of diagnosis there are high rates of grade 2 disability amongst persons with leprosy and 100% of persons with confirmed Buruli ulcer present with ulcerative lesions [64]. There is a growing evidence base from other settings regarding co-morbidity between skin NTDs and mental ill-health [6,7], and an apparent co-occurrence of these two epidemics within Liberia.

This epidemiological evidence thus suggests co-morbidity between NTDs and mental distress, but further study of biological interactions would be required [12]. Our analysis shows that the historic and social context of war in Liberia contributes to the production of multiple ‘generalised stressors’ or risks (Fig 5) that promote the existence of these co-occurring epidemics [34].

We used NTDs as an entry point to explore the relationship between these co-occurring epidemics; however using mental health as an entry point in encouraging participants to share their stories could also support in understanding if the interaction between these two epidemics is bi-directional [3]. Data presented here can only support a unidirectional interaction by which chronic morbidity and disability associated with NTDs (particularly poor access to information and care) contribute toward periods of mental distress, catalysed by experiences of stigma in the context of structural violence. However, other research studies in Liberia and elsewhere in sub-Saharan Africa have shown that delays in generalised health seeking as a result of mental ill health are common due to stigma, financial constraints, and lack of knowledge of illness [60,65,66]. Lack of management of mental ill-health has been described as exacerbating these delays; this is common in Liberia due to ongoing health systems weaknesses [60,61] (further discussed below). Thus, based on shared environmental risk factors and micro level stressors, including dual stigmatisation, it is likely that these co-occurring epidemics may have a bi-directional interaction. This would align with evidence from other syndemics where depression is frequently cited as a key element that can worsen health and wellbeing outcomes of chronic illnesses [1].

### How are the conditions experienced by affected persons and their social networks?

As suggested by Medenhall *et al* (2017) within syndemics *‘culture shapes meaning associated with suffering and social responses to suffering, illness and disability’ [2]* (p953). Within this section, we draw on and adapt Meyer’s (2003) ‘minority stress model’ [34] (see Fig 5) to emphasise the critical role of stigma in promoting the synergistic interaction between NTDs and mental distress. Firstly, we suggest that the post-conflict environment presents multiple ‘generalised stressors’ as a result of structural violence that may contribute to periods of mental distress. For people affected by NTDs (who we argue have a ‘minority status’ due to experiences of disadvantage) [34], we hypothesise, based on our data, that these ‘generalised stressors’ are exacerbated. However, the degree of exacerbation is also dependent on the impact of intersecting power relations such as gender, generation and geography that shape individual identity and resilience. As has been found in relation to other chronic conditions, we specifically consider how the mutually reinforcing interactions between somatic experience, ability to complete daily activities and the social meaning of disease (social isolation) can shape experience [2]. Secondly, we consider how both ‘external and internal minority stressors’ [34], specifically varying processes of stigmatisation and discrimination, become additive to ‘generalised stressors’ to produce an excess risk of mental ill health amongst people affected by NTDs due to social stress which is resultant from social and structural inequities [34]. Finally, by considering how both ‘generalised’ and ‘minority stressors’ become embodied in different ways based on intersecting axes of inequity such as gender, generation and geography we can consider how internalisation of oppression functions in different ways to shape nuanced experiences of the syndemic.

### General stressors

The following three sub-sections explore descriptions of how experiences of general stressors emerging from narrative accounts were shaped and exacerbated by minority status and identity and contribute to the impact of negative health outcomes.

#### Pain, poverty, precarious livelihoods and financial stress

Physical limitations associated with NTDs have been described as a key pathway for their contribution toward poor mental health due to lost job opportunities, and dependency on others [67–69]; however, this has infrequently been considered in relation to its impact on gendered social roles and the broader economic environment. Within narrative accounts, precarious livelihoods that centred around day to day survival (often a combination of strategies including subsistence farming, selling, and fishing), coupled with individual’s inability to fulfil expected gendered social roles (e.g. as a wife, mother, husband or father) were a key focus and trigger of stress. Somatic experience, including variations in pain and functional limitation experienced at different time points, and changes in physical appearance, contributed to the impact of illness on livelihoods. For some, restrictions in mobility and alterations to physical appearance were permanent whilst others described these as more transient or temporary and triggered by specific activities or social situations.

For women and men affected, the degree of independence in completing livelihood activities was a key determinant of the level of stress experienced. Many individuals would struggle through daily activities to reduce negative impacts on the household and to complete their role as a wife, mother, husband or father. For example, for persons affected by BU, particularly women, having to complete livelihood tasks that involved use of the affected area or limb frequently triggered pain and mobility restriction. Alice, who had ulceration on her legs, *‘had no one to do things for me’* and so *‘when I was walking long distances*, *I can feel [pain]…but I have to force it to get my daily bread’* **(CS017, Female, BU, Nimba).**

For some people with lymphoedema, restricted mobility linked to physical impacts of disease and associated stresses was explicitly linked to the experience of acute attacks. During acute attacks, the impact on households and associated stresses were exacerbated, particularly when participants felt gendered roles and responsibilities within the household were compromised. For example, Natt, described that during these periods there was a heavy load on his wife; he found this particularly difficult and demasculinising as he was not able to fulfil his role as a husband during these periods and it is not what should be expected of him *‘at his age’* **(CS008, LF, Male, Maryland)**. By subconsciously trying to live-up to patriarchal advantage and fulfil a position of ‘hypermasculinity’, which is highly resonate in Liberia as a result of protracted conflict [60], both Natt and Jon experienced stress or isolated themselves socially as a coping mechanism. However, as particularly exemplar in Natt’s case this stress was not static; rather, the interplay of disease severity and ability to fulfil gendered ideals mattered. For Jon and Hannah, and others alike, protracted negative impacts on their livelihood activities coupled with social isolation and stigmatisation had resulted in extremely poor living conditions, leaving them vulnerable to additional health and social risks, including for Hannah fear of gender based sexual violence.

#### Stress, social isolation and challenged gendered identities

In some cases, for example in Jon’s story (Fig 3), inability to complete tasks ‘expected of a man’ had led to men being left by their spouses and isolated from children and other family members. This appeared to be less linked to illness origins and more to the severity of illness and/or permanency of impairment. Conversely, the onset of illness had caused some men to worry that they may not be able to find a spouse because of illness. However, this was often not the case, with some men marrying and having children post illness.

Many young women, had similar experiences to those described by Hannah (Fig 4) and had been left by an intimate partner, or approached for sex and then abandoned when they became pregnant. For some women, particularly those living with lymphoedema and onchocerciasis this was a repetitive pattern, where men would approach them during periods where the ‘leg was small’ however following acute attacks they would be abandoned. For Hannah, and others alike, desire to fulfil gendered identities of motherhood frequently led them to make choices which presented them as vulnerable to inter-personal relationships that were challenging; this emphasises how social constructions of gender can influence women’s agency in decision making [27].

Women who also constructed their identity around motherhood, felt a sense of sorrow as they were often unable to care for their children as they would have liked and therefore many children were living with other relatives or, in some cases, mothers felt they were not receiving the care they needed. Social isolation because of illness particularly from the natal family was linked in narratives to a shift from being able to contribute to household livelihoods to becoming a dependent. This was particularly true for male participants, suggesting a link between prescribed gender roles and social isolation coupled with challenges to economic circumstance.

*‘if I complain about the sore they will feel that I want money from them…*. *I feel bad*, *we supposed to be together*, *joke together*, *play together as a human being*…***(CS026*, *Male*, *BU*, *Bong)***.’

#### Health stress, social and structural barriers

Jon and Hannah’s stories are illustrative of many narrative accounts that emphasise the stress that barriers to health care access brought to participants both during the conflict and more recently. As described in these two stories, failure to access essential services due to conflict, lack of financial resources, and stigmatisation by health workers was often seen to result in life altering physical morbidity.

Within narratives where participants had become sick post-conflict, multiple care seeking visits were a dominant narrative thread and source of stress, with participants describing ongoing oscillation between formal and informal health providers in constant search of a diagnosis or cure for an often unknown disease condition. Being unable to understand the cause of their impairment or poor physical health was often described as distressing for participants [46]. Repetitive health seeking was often necessary as a result of weak health system capacity to be able to diagnose or identify cases due to: poor knowledge amongst health care personnel; limited supplies and weak laboratory systems that restricted health worker ability to confirm cases and initiate treatment, particularly for BU; and a shortage in medicine supplies, especially when medicines are not freely donated without laboratory confirmation. Where medicine supplies were limited, health workers reported having to make decisions about who to provide medicines to, rendering some individuals without access to treatment, thus exacerbating morbidity.

*‘sometimes you have four or five cases, sometimes you will only have treatment for two person and the drugs are not on time…’ **(KII006, County NTD Focal Point)***

The necessity of repetitive health seeking brought further financial stress and strain to individuals and households and many individuals resorted to seeking alternative care, predominantly within the ‘traditional health system’. The traditional health system was also frequently cited as a first point of call for many people due to their syncretic perception of disease origins not considered in the formal health system, and a trust in the traditional system because of community proximity and stability during periods of fragility [30].

Stress associated with precarious livelihoods, social isolation and weak health systems infrastructure represents a conflux of political-economic and social inequalities. Particularly evident within narrative accounts of stress was the ways in which individuals have internalised the social domination to which they are subjected based on gendered identities [27]; this process has been described as symbolic violence [27] due to its contribution toward poor health outcomes. By applying framings of both structural and symbolic violence, coupled with intersectional thought, we can consider how general stressors (see Fig 5), as a result of social and structural inequities, become embodied in multiple and alternate ways to shape unique and contextualised experiences of syndemic suffering.

### Stigma: Internal and external minority stressors

In the subsequent three sections we discuss ‘types’ of stigma identified within the framing of Scambler’s hidden distress model of stigma [70]. This presents critical distinctions between stigma which is actually experienced (‘enacted’) and that which is ‘felt’ (either as a result of anticipation of enacted stigma or that which becomes internalised) [70,71]. These experiences become a product of the wider social and structural context in relation to health and illness and can be described as internal or external ‘minority stressors’ [34] (see Fig 3). We argue that it is these ‘minority stressors’ that exacerbate or are additive to ‘general stressors’ as stigma associated with these illnesses, coupled with the stigmatised identities of people affected by them, become a critical driver in shaping the interaction between mental distress and NTDs in Liberia [49].

#### Enacted stigma (external minority stress)

Stigma was commonly described as a reason for experiences of violence and as has been documented in other settings, was a driver of mental distress associated with NTDs [7,71,72]. Mary (CS018) described that her experience of enacted stigma (through verbal insults) in the community made her husband very angry and led him to leave her at the leprosy hospital. Although she described her husbands’ actions as based in love for her, this revealed a lack of autonomy or control by Mary in decision making, based on underlying gendered powered hierarchies, which contributed towards her experiencing emotional distress and isolation. Stigma in Mary’s case became a key driver through which leprosy and mental distress became interconnected and mediated through her identity as a woman to threaten her health and wellbeing.

*‘My husband, he sent me to the colony…he never gave me any cross word; but people who live in the community they are the people who were insulting me. They made the man vex (angry) to say ‘‘I will take my woman to medicine…”. Then at that time then I began to cry. He said, “don’t cry I will send you to medicine man; I will carry you.”‘ **(CS018, Female, Leprosy, Nimba).***

Case study narratives related to leprosy and BU, revealed a complex relationship between in-patient treatment, religion, enacted stigma and psycho-social well-being. For many, in-patient treatment as a result of being around other patients provided a sense of comfort and social support, and where in-patient treatment was provided through faith-based organisations religion often provided additional relief. Regardless of treatment location, faith was particularly pivotal for affected persons during periods of depression or when experiencing suicidal thoughts, as the illness was felt to be part of *‘god’s plan’*.

*…when your life story change*, *it looks bad…I even decided to kill myself but then God just made a way…That’s how my life became*…*I used to think about killing myself; now*, *I say it was good I didn’t do anything to myself because that’s god will’* ***(CS012*, *Male*, *Leprosy Patient)***.

Despite the positive benefits of in-patient treatment and the critical role and introduction of faith as a coping mechanism for many affected persons, both historic and ongoing in-patient care had impacts on the ability of some leprosy and BU patients to re-integrate with their communities. Some persons affected by leprosy narrated that even when they had completed treatment and were no longer infectious, the community would still see them as sick, feeling that; *‘even if we received treatment for eight years*, *nine years*, *leprosy can’t finish (CS009)*’. Isolation and rejection from their communities often had significant negative impacts on affected persons mental well-being.

#### Hereditary stigma (external minority stress)

Hereditary stigma [71,73] was also commonly experienced, frequently leading to social isolation of the household from the broader community. Joy’s sister described how since her sister had returned from in-patient treatment for leprosy her ‘*own friends don’t want to come here [to the house]’* ***(CS011*, *Nimba)***. Princess also described similar experiences of hereditary stigma within inter-personal relationships that meant she now kept her father’s illness to herself where possible.

*‘because they [my friends] didn’t know my father’s condition, nothing bad they can tell me to make me feel bad [they are nice to me]. But because the man…the man who can asked me where my father is, and I told him about my father…the problems can be there [he is unkind because he knows about my father]…’ **(CS012, Princess, Nimba).***

Threats to inter-personal relationships for many women in relation to some NTDs has been considered elsewhere [67,74], however the impact at household level is seldom described. Where multiple people within one household were sick, social isolation and separation were exacerbated. However, for older participants, stigmatisation and associated isolation was described to a lesser extent based on the perception that getting sick in old age was seen as inevitable.

#### Internalised or anticipated stigma (internal minority stressors)

Many participants, particularly women, although some men, also showed signs of internalised or anticipated stigma [71,73], describing that they felt ashamed to expose body parts in public. These behaviours have been described elsewhere in relation to NTDs, particularly when impairment was acquired in adulthood based on the fact that prototypes of health and normalisation were often learned from a young age [35]. This was also true within our study. Some women described wearing longer ‘lapper’ to cover limbs so that they weren’t exposed or visible when moving around in public, men described wearing ‘*loose and long trousers*, *so that people won’t know until they open it’* **(CS026, Male, BU, Bong)**.

As well as adjusting their behaviour, many participants described that they would *‘sit and think’* or be *‘thinking too much’* as a result of physical morbidities and social responses to their disease condition. Many participants also described withdrawing themselves socially as a result of stigmatisation, and a few participants also described a dependency on alcohol during periods of isolation as a coping strategy, thus presenting them with further vulnerabilities to additional risks such as violence.

*‘Myself when it happened to me I was thinking too much so I nearly I was a drunk on the street, I will not lie.’ **(CS012, Male, Leprosy, Nimba)***

Phrases such as *‘thinking too much’* have been identified in other sub-Saharan African settings as being ‘idioms of distress’ used to communicate psycho-social ill-health [75]. A study in Maryland, Liberia, identified two categories of idioms of distress; those related to the mind and brain and those related to the heart [76]. The authors present ‘*thinking too much’* as a brain idiom that is closely related to heart related terms but which extends beyond everyday experiences of sadness to describe mental distress that can lead to stigma and discrimination [76]. Descriptions of these idioms of distress within narrative case studies suggest evidence of co-occurrence of NTDs and mental distress. Stigmatisation (enacted, hereditary, internalised and anticipated) [71,73] plays a critical role in driving the interaction between biological and social and structural processes or stresses that threaten health; thus indicating evidence of a stigma syndemic [49].

By facilitating an analysis of the ways in which stigmatisation intersects with factors that shape individual identity (e.g. gender and generation) to form varying pathways of bio-social interaction, intersectional theory has the potential to significantly add to the existing literature on stigma syndemics. Understanding how intersecting power relations shape the way that stigma becomes transformed from a social phenomenon into a force that can create devastating health outcomes is critical in being able to shape adequate social and health systems responses [34,49]. Some argue that the role of stigma in illness experience linked to NTDs is overplayed often at the expense of other factors that can lead to social isolation and consequent mental distress [77]. However, as has been highlighted in relation to other syndemics that are promoted through structural and social processes of stigma and discrimination [49], we propose that the experience of stigma in relation to NTDs and mental distress may warrant consideration within a syndemic model in other settings.

### How can health and social systems contribute to responses?

We have shown how health systems failures and social and structural inequities contribute to experiences of syndemic suffering. Within the following section we focus on the implications of syndemic analysis for ways forward. Liberia’s health system is undergoing a period of reform and national priorities reflect a shift toward more integrated health service delivery to ensure efficient use of scare resources [78]. Integrated, people-centred health systems are increasingly recognised as more effective in responding to the health needs of populations, particularly those in low income settings, and draw on ideals of ‘syndemic care’ by ensuring the advancement of treatments and interventions that respond holistically to broader social and structural inequalities that shape disease interactions and create ill-health [2,13,79]. Thus, person-centred approaches that are grounded in syndemic understandings of disease and illness offer an optimal framework for informing the type of multi-level responses that are required to improve individual and population health outcomes in Liberia. A shift in thinking towards more person-centred approaches within NTD control is reflected within the recently released NTD roadmap- ‘*Ending the Neglect to attain the Sustainable Development Goals. A roadmap for neglected tropical diseases 2021–2030’ [80]*. Within the following sub-section and summarised alongside our key findings within Table 3; we aim to make suggestions of practical actions that NTD programmes could take to support them on their journey toward person centred care that addresses the holistic (including mental health) needs of persons affected by NTDs.

**Table 3:** Overview of findings and recommendations for NTD programmes.

Summary of Key Findings	Programmatic Recommendations (Informed by findings and literature)
Co-occurrence of epidemics of NTDs and mental distress in Liberia.	Enhance co-ordination and collaboration between health and social care professionals focused on mental ill-health and chronic conditions at co-ordination levels within the health system (national and county). For example, through establishing integrated case management teams.
Post-conflict environment leads to generalised stressors (**pain, poverty, precarious livelihoods, financial stress**) that intersect with gendered identities to shape experience, impact and meaning of illness, chronic morbidity and disability. Specifically, when catalysed by **stigma** (enacted, hereditary, internalised/anticipated) can lead to **periods of mental distress** (including feelings of helplessness worry and anxiety leading to suicidal thoughts and or attempts), shaping the **syndemic relationship.**	**Community Awareness Campaigns** to promote stigma reduction [81,82] **Community-based participatory learning programmes** are required to redress social inequities and to create an enabling environment for people affected by NTDs. This is particularly critical in minimising the contribution of power hierarchies in shaping negative disease interactions (e.g. between NTDs and mental distress). **Stepping Stones** [83], is one example of this. **Patient advocates** to promote self-care to reduce the impact and frequency of acute attacks [82] **Peer-support groups** that prioritise the management of chronic pain. **Early case detection** through active case finding to limit risk of reduced positive prognosis. Consider applicability of **socio-economic rehabilitation to contribute towards stigma reduction** through the provision of: • skills training • enhancing entrepreneurship • educational opportunities **Provision of specialised rehabilitation or habilitation services** including the provision of assistive devices. Use of ICF as a tool to design rehabilitation programmes to explore the interactions of functional limitation and environment leading to participation barriers [84].
As a result of social and structural inequities, people affected by NTDs face multiple **participation restrictions**, including: • In **decisions to seek care**- authority of health care providers (clinicians and country doctors), age and gender shaped individuals’ power in care seeking • Being **unable to attend school-** leading to feelings of loss and social isolation • Being **unable to fulfil gendered ideals-** men feeling unable to fulfil livelihood activities led to challenged masculinities; for women transition to ‘dependent’ and inability to care for household led to feelings of worry and anxiety	**Patient Advocates** to support in identification of affected persons and in care delivery [85]. Patient advocates could also be utilised to support community reflection on gender roles and norms.
**Health Seeking Delays** are exacerbating morbidity, disability and associated stigma as a result of structural inequities. **Further mental distress and NTDs impede care seeking for each other**. Barriers include: • **Poor Quality of Care**: Lack of skilled health workforce available for diagnosis or mis-diagnosis; stigma experienced by affected people from health workers • Weak supply chains leading to a **shortage of medicines** and inability to meet increased demand • **Limited absorptive capacity** of the health system due to limited human resources and minimal focus on systems strengthening • **Limited understanding of disease severity** • **Pluralistic belief systems** led to a web of complex care seeking • **Conflict** • **Financial Barriers** • **Absence of referral** between NTD programme and mental health clinicians.	**Active Case Searching:** • Consider the use of community health cadres whilst also maximising use of evidence on managing performance [86,87] with a specific focus on implications for equity. • Align activities to ongoing community health systems reform in Liberia. • Patient Advocates to support case finding [85]. **Improve Diagnostic Pathways and Processes** by: • Piloting application of easy to use clinical diagnostic tools with primary health care workers e.g. WHO algorithm for stigmatising skin disease; SkinApp • Implementation research designed to strengthen laboratory system and supply chain for rapid tests and microscopy as well as PCR testing. Explore strategies for **decentralisation of mental health service provision**, for example through the use of: • Friendship benches [88] • Community-based participatory learning dialogues (see above) **Strengthen referral pathways at primary and secondary treatment level to MhGAP trained clinicians** who are able to provide ‘talk therapies’ to affected persons.
**Key Coping Mechanisms Included:** • Reliance on religion and faith in navigating trauma, rationalising experience and practicing forgiveness leading to a renewed sense of purpose or belonging within the community. • Identification of safe spaces (e.g. settling around leprosy treatment centres) or individuals in the community to seek guidance and support. • Interactions with other people affected to support in processing illness experience.	**Peer Support Groups** to support with medical management e.g. washing and wound management [82], but also to improve self-respect and community participation [89]. **Improve co-ordination and collaboration with pluralistic service providers**. Participatory dialogues could be facilitated to enable this collaboration to support social support and early case detection. Support groups that span chronic disease conditions should be considered.

Our findings emphasise that the social world of our participants cannot be disconnected from their experiences of NTDs and mental distress; thus, health systems must be able to respond to both the social and psychological dimensions of people’s lives to be able to successfully manage NTDs [27]. By using a syndemic framework to consider how social and structural stressors shape impacts on health, it is apparent that NTDs and mental distress in Liberia require multi-sectoral responses, which require further investigation and research. This is also a key priority area identified for future advance and action within WHO’s NTD Roadmap 2021–2030 [80]. Intervention approaches that focus on the cause of suffering, such as by addressing stigma or livelihood needs are essential in improving the health and wellbeing of affected populations in Liberia. ‘Stepping Stones’ is one example of a gender transformative community-based participatory learning programme focused on HIV that aims to minimise the contribution of power hierarchies in shaping negative disease interactions (e.g. between HIV, Gender Based Violence and Mental Distress) and stigmatisation [83,90]. By focusing on community norms and values, such approaches are in line with people-centred responses to ill-health as they are adaptive to the contextualised experience of suffering at the community, household and individual level. Participatory processes of adaptation and learning similar to ‘Stepping Stones’ could support in the design and delivery of ‘syndemic care’ [2] in relation to NTDs and mental distress in Liberia.

Putting people and their values at the centre of health intervention design is a key underlying principle in people centred health systems [91]. Intervention responses must therefore be mindful of medical syncretism and pluralistic health seeking, especially since our study supports other studies in Liberia emphasising that the traditional health system is widely consulted during periods of psycho-social distress [30,92]. The current absence of collaboration between the traditional and formal health system in relation to NTD management delays health seeking and exacerbates physical morbidity. Despite challenges of collaboration with the traditional health system in other sub-Saharan settings due to the ‘paradigmatic disjunctures’ and uncertainty between varying treatment approaches, successful partnerships have been achieved in the presence of mutual respect and through collaborative intervention design [93]. Actors in the traditional health system could be important partners in responding to the syndemic interaction between NTDs and mental distress in Liberia.

Prolonged conflict and post-conflict fragility have resulted in an inaccessible and under-resourced health system in Liberia, with very limited specialist services. As a result, two key challenges remain in providing responses and treatment for synergistic interactions between NTDs and mental distress; 1) identifying patients and 2) health workforce limitations. Tasking and training community health volunteers to support early case identification is a key strategy to mitigate this barrier to diagnosis within integrated case management plan and has proven to be effective for BU in other endemic settings [86]. However, as with many community health interventions, key Informants described ongoing challenges in the referral of patients due to accessibility barriers (geographical and financial), stigmatisation and attrition of community health volunteers due to lack of incentives in the context of precarious livelihoods. Expanding the reach of health systems to rural areas and better utilisation of the evidence base [87] on maximising the performance of community health volunteers could strengthen the role of this cadre in early case detection for NTDs.

Health personnel described a key health systems weakness as an absence of referral between the NTD programme and mental health clinicians at point of diagnosis. Both NTD and mental health specialists suggested that the introduction of mental health screening and counselling at point of diagnosis accompanied by a strengthening of referral between these departments would strengthen responses to patient support needs. Our analysis concurs with Litt *et al* (2012), who see ‘mainstreaming mental health support within NTD treatment’ as a critical step in meeting the psycho-social support needs of affected populations [7]. However, key informants identified human resources capacity gaps at the primary and secondary health care level as likely hinderances to cross-departmental collaboration and referral. By taking a syndemic approach to care at the community level, through the provision of integrated (non-disease specific) psycho-social support interventions, these capacity gaps have been addressed in other sub-Saharan African settings. For example, ‘community friendship benches’ [88] that train lay community workers to deliver counselling services have been shown to alleviate symptoms of depression [88] in Zimbabwe. Capitalising on these approaches could support in fostering a more holistic response to the health needs of persons affected by NTDs in Liberia.

## Discussion

Syndemic relationships have been most clearly articulated in relation to HIV [2]. However, advancing syndemic theory and responding effectively to the health needs of populations within LMICs, requires application of syndemic theory to other disease conditions [2]. Our findings suggest that there is a specific need for further exploration of the syndemic relationship between NTDs and mental distress in the context of structural violence (post-conflict fragility, poverty, gendered social norms, stigma, and weak health systems infrastructure). We have argued that ongoing social inequalities and the broader political economy in Liberia, create a perpetuating cycle of negative health and social outcomes for those affected by mental distress and NTDs that will require multi-level systems responses. Intersectional theory has allowed us to consider how structural violence becomes embodied in different ways through interacting multi-level processes that shape experience according to social location. Thus, intervening to transform the social, environmental or political factors that contribute toward the interactions between health conditions is essential in minimising burdens of ill-health associated with NTDs and mental distress [2].

The utilisation of an unstructured methodology in the form of narrative case studies, allowed affected persons and their families to guide the narrative direction and prioritise what was important in sharing their reality. Unstructured qualitative methodologies such as these can be useful in advancing syndemic theory and in exploring syndemic interaction in other contexts, as they provide deep understandings of the mechanisms that lead to syndemic relationships [3,94]. The power of experience in guiding our exploration of syndemic suffering was essential to be able to fully understand its complexity; by taking an intersectional approach to analysis we were able to better explore how lived experiences cannot be separated from larger socio-political contexts that shape people’s ways of being in the world [27]. We propose that it is the critical analysis of experience that enables the development of appropriate people-centred responses to syndemic suffering, which, in striving to redress existing social and structural inequities is more useful than quantitative analysis in isolation.

Although there is clear strength in the in-depth analysis presented here, it should be acknowledged that such an approach was highly labour intensive (for both researchers and participants), which may present challenges in future studies if not adequately considered. We believe the longevity of the study is what generated such rich and nuanced understandings from participants; as establishing trust and rapport to gain honest and transparent accounts was critical. Finally, although narrative methodologies can be therapeutic [46], ensuring that adequate ongoing support mechanisms are in place for study participants is of ethical imperative but can be challenging within under-resourced and overstretched health systems.

Evidence of systems weaknesses, including poor human resource capacities, inadequate diagnostic infrastructure and absent referral pathways, present a need for the development of high-quality integrated approaches for the management of NTDs in Liberia [95–97]. However, ‘integration’ within health systems discourse has predominantly focused on merging and co-ordinating disease control activities within health-care delivery [98], with limited consideration of the biosocial interaction of disease. Our study presents a critical need to bring together health systems and syndemic discourses to broaden the focus of integration to consider ‘syndemic care’ [2,13]. Syndemic care approaches within health systems development would not only maximise resource use but allow for the consideration of the biosocial context of disease and the development of interventions to transform this.

## Supporting information

S1 Text Case study summaries. (DOCX)

S2 Text Inclusivity questionnaire. (DOCX)
