# Supplementary material for: A syndemic born of war: Combining intersectionality and structural violence to explore the biosocial interactions of neglected tropical diseases, disability and mental distress in Liberia
Source: PLOS Glob Public Health. 2022 Jun 29;2(6):e0000551. doi: 10.1371/journal.pgph.0000551 (PMC10021464; doi:10.1371/journal.pgph.0000551)
Supplement: S1 Text — (DOCX) [file pgph.0000551.s001.docx]

**Supplementary File 1: Case Study Context**

Before providing a summary of each of the case studies that informed data presented, the below table provides a summary of case study demographics.

| **Case Study Number** | **Age** | **Sex** | **Marital Status** | **Occupation/Job Role** | **Educational Level** | **Disease of Interest/Clinical Manifestation** |
| --- | --- | --- | --- | --- | --- | --- |
| **Maryland County** | | | | | | |
| **CS001** | | | | | | |
| Illness Narrative | 25-49 | F | Widow | None | No Education | Buruli Ulcer (arm) |
| **CS002** | | | | | | |
| Illness Narrative | 25-49 | F | Partnered | Market Women | Attended Primary School | Lymphedema (Severe, Left Leg) |
| **CS003** | | | | | | |
| Illness Narrative | Over 49 | M | Widow | None | Noe Education | Lymphedema (Severe-left leg; moderate-right leg) |
| **CS004** | | | | | | |
| Illness Narrative | 25-49 | F | Married | None | Attended Primary School | Lymphedema (moderate-right leg; mild-left leg) |
| **CS005** | | | | | | |
| Illness Narrative | Over 49 | F | Widow | None | No Education | Leprosy |
| **CS006** | | | | | | |
| Illness Narrative | 25-49 | M | Single | None | Attended Secondary School | Hydrocele |
| **CS007:** Excluded at participant request | | | | | | |
| **CS008** | | | | | | |
| Illness Narrative | 25-49 | M | Married | None | Attended Primary School | Lymphedema (Severe-right leg) |
| **Nimba County** | | | | | | |
| **CS009** | | | | | | |
| Illness Narrative | 18-25 | M | Married | None | No Education | Leprosy (discharged) |
| **CS010** | | | | | | |
| Illness Narrative | Over 49 | M | Single | Farmer | Attended Secondary School | Leprosy (discharged) |
| **CS011** | | | | | | |
| Illness Narrative | Over 49 | F | Widow | None | No Education | Leprosy (discharged) |
| **CS012** | | | | | | |
| Illness Narrative | Over 49 | M | Partnered | Night Security | Attended Secondary School | Leprosy (discharged) |
| **CS013** | | | | | | |
| Illness Narrative | 18-25 | F | Partnered | At School | At School | Buruli Ulcer (discharged) |
| **CS014** | | | | | | |
| Illness Narrative | 25-49 | F | Single | None | Attended Secondary School | Onchocerciasis (Blind) |
| **CS015** | | | | | | |
| Illness Narrative | Over 49 | M | Married | Farmer | Attended Secondary School | Onchocerciasis (Blind) |
| **CS016:** Excluded non-NTD case study, only became apparent during narrative. | | | | | | |
| **CS017** | | | | | | |
| Illness Narrative | 25-49 | F | Single | Fufu Seller | No Education | Buruli Ulcer (discharged) Leprosy (discharged) |
| **CS018** | | | | | | |
| Illness Narrative (NB: wife of illness narrative CS012) | Over 49 | F | Married | None | No Education | Leprosy (discharged) |
| **CS019** | | | | | | |
| Illness Narrative | 25-49 | M | Single | Farmer | No Education | Leprosy (on treatment) |
| **CS020** | | | | | | |
| Illness Narrative | 18-25 | F | Single | Market Women | Completed Primary School | Leprosy (on treatment) |
| **CS021** | | | | | | |
| Illness Narrative | 18-25 | M | Single | At School | At School | Leprosy (on treatment) |
| **CS022** | | | | | | |
| Illness Narrative | 25-49 | F | Partnered | Selling | Attended Primary School | Leprosy (on treatment) |
| **CS023** | | | | | | |
| Illness Narrative | 25-49 | M | Partnered | Farmer | No Education | Leprosy (on treatment) |
| **Bong County** | | | | | | |
| **CS024** | | | | | | |
| Illness Narrative (NB: niece of illness narrative CS025) | 18-25 | F | Single | Market Women | Attended Primary School | Buruli Ulcer (on treatment) |
| **CS025** | | | | | | |
| Illness Narrative | 25-49 | M | Married | None | Attended Secondary School | Buruli Ulcer (on treatment) |
| **CS026** | | | | | | |
| Illness Narrative | 25-49 | M | Single | Selling Books | Attended Primary School | Buruli Ulcer (on treatment) |
| **CS027** | | | | | | |
| Illness Narrative | Over 49 | F | Widow | None (retired) | None | Onchocerciasis (Vision Impairment Skin Complications) |
| **CS028** | | | | | | |
| Illness Narrative | Over 49 | M | Married (wife left) | None | Attended Secondary School | Onchocerciasis (Blind) |

The following case study summarises are shortened from accounts developed during the analytical process to ensure the protection of participant identity.

**Maryland Case Studies**

**Case Study Summary CS001**

**NB: This case study was collected at the main referral hospital in Maryland County. The patient had come to speak to us and the interview was completed in the NTD Focal Points office. As a result, we were unable to speak to any other members of the patient’s household.**

Female aged 26-49 years old living with Buruli Ulcer. Widow. Clear ulceration on right arm, which had waisted, and she was no longer able to straighten. The NTD focal person described that he was unsure that she would be able to straighten the arm again or at least was unlikely to resume full movement even if the ulceration was treated. The participant described a complex care seeking pathway having been resident in Ivory Coast and trying to seek treatment there, following a confusing and unsuccessful treatment process, where there was suggestion of arm amputation, she had decided to move back to Liberia to seek treatment. This decision was influenced by her mother. Her sister had left her family and moved to Liberia with her to support her in seeking treatment and in household activities. She described here sister as her main caregiver. Her three children had also moved back to Liberia with them. The participant was known to the NTD programme, and regularly travelled a long distance from her community to the main referral hospital in search of treatment. The NTD focal point expressed frustration that they had been unable to secure medicines from the central level to treat this patient despite constant interaction with the national NTD programme, highlighting bottlenecks in the drug supply chain. The participant was particularly concerned about the impact the illness was having on both her sister and her children (who she could no longer pay for education for).

**Case Study Summary CS002**

Female aged 25-49, living with severe lymphedema in the left leg. The symptoms began when she was a child in 1995 during a period of conflict. Her father carried her to the main referral hospital in Maryland county where on her second trip she was told she had a worm inside her. Due to the sickness she stopped going to school. This case study was completed in Maryland county. The patient had lived here her whole life. We also spoke to her two brothers as part of the case study interviews. Both brothers described feeling worried for their sister as well as fearful that they may also catch the sickness. One brother was physically disabled as a result of a road traffic accident.

**Case Study Summary CS003**

Male aged 68 years old living with lymphedema in both legs (right severe and left moderate) and hydrocele. Participant identified his sister as main caregiver/person of importance that we should also talk too. Participant has six children, two have passed away and the other four live far away/left. He is a widower, wife died in 2004, before the sickness (began in 2007). The participants sister also talked of not being in excellent health, describing living with a chronic cough and having previously been diagnosed with TB. She is also a widower. Both described having no education and no fixed form of occupation or income. Case study was taken within Maryland county, participant described moving back here from Grand Cedeh during the conflict. ‘*He said the war, the 1990 war crisis came that what brought him down, he and his children’.*

**Case Study Summary CS004**

Female aged 25-49, living with lymphedema that could be described as moderate in the right leg and mild in the left leg. Her and her husband, who was the other person interviewed as part of this case study, have one child. They described a want for another child, but had been unable to conceive, the husband within the case study attributed this failure to conceive to the ‘big foot’ for which they were seeking treatment. The participant was married to the town commissioner who she had met after the illness began. Throughout the interview, the husband appeared very supportive of his wife, expressing that she had the sickness before they met so this could not shape the way he felt about her. The housing and appearance of the family home and setting indicated that there were relatively better off than others in the community, perhaps because of his job role. The illness began when she was in 3^rd^ grade which led her to drop out of school, she described feeling a sense of loss linked to this, which was frequently triggered when she saw children in school uniform.

**Case Study Summary CS005**

Female, over 49, who had been diagnosed with leprosy and had lesions on both legs. She was a widow and living with her aunt and her husband, who were her main ‘caregivers’. She had been living with symptoms of leprosy for about one year. She described having been a farmer her whole life which led her to move around to make money to be able to send her children to school. When the leprosy started she had been living in a different town, however her aunt brought her back to Rock Town when she got sick, which is where she was born. We spoke to her nephew to complete the case study as this was who she identified we should interview. Her nephew described economic hardship in the household and that they were very reliant on their farm produce to survive. He described the lack of having a ‘big job’ as due to the fact that his father was blind, which had caused him to leave school without graduating in 1987. She described ‘taking tablets’ that had been prescribed to her by the Rock Town clinic and having to return for more of these tablets on a monthly basis. When asked what the tablets were for, she said that she wasn’t sure, but knew they were for her foot problem.

**Case Study Summary CS006**

Male, aged 25-49, living with hydrocele, unmarried. The sickness started two years ago. The case study was taken in Maryland where the participant was born and lives. The participants brother was the other person interviewed as part of the case study. Prior to getting sick the participant was a fisherman and would fish daily leaving from the community between 7am and 4pm. He had sought treatment at the hospital several times for the illness and was told to return for an operation in June. The participant described having gone to school until secondary school level but had dropped out in 11^th^ grade due to being unable to afford school fees. He had travelled to attend secondary school in Plebo and Harper (main towns in Maryland county). His brother described a fishing accident that had injured his brothers’ hand and following which he hadn’t been the same since, he sounded like he was describing some elements of PTSD or depression associated with the event. Hydrocele in this case, could be seen as a co-morbidity with pre-existing mental health challenges.

**Case Study Summary CS007:** excluded at participant request

**Case Study Summary CS008**

Male, aged 25-49, living with severe lymphedema in right leg. Participant is married, and his wife was the other person interviewed as part of this case study. They are living in Maryland, and have had three children, but one has died. The illness began in 1994 before they were married or knew each other. This was during the war time, ‘*where there were no medicines available’*, and so he had to seek medicines from the country doctor in exchange for the payment of a cow. The country doctor was based in Ivory Coast and was seen by the participant as their only option during this time. This treatment involved cutting in the skin. Since that time, the participant has taken many medicines for pain management, predominantly Panadol. He described leaving school because of the sickness and was unable to have the ‘upper hand’ unlike some of his siblings.

**Nimba Case Studies**

**Case Study Summary CS009**

Male aged 18-25 years old, living with the effects of leprosy, specifically gnarling on left and right foot. Treated as an inpatient at leprosy treatment centre and returned to the community. He had been given shoes at leprosy treatment centre to aid him in walking. Participant identified his wife as the other person that we should talk to as part of the case study. She was 25-49 years old and living with leprosy. The case study was completed in Nimba county, in Lepula community. The patient had lived here his whole life, whereas his wife had moved to the community with her mother because of her marriage. They had then met and married before the leprosy had developed. Together they have had five children, but the officer in charge of the health facility informed us that each time she gives birth the family and community describe that she is too sick to look after the baby so they take the baby from them, each time the baby has subsequently died (they think because of a lack of milk etc). Together they both experienced high levels of stigma and social isolation. They both seemed to crave social interaction and a want for friends and social acceptance. The patient had a dog that he was very attached too and went everywhere with them.

**Case Study Summary CS010**

Male, aged over 49, has problems with his feet and gnarling on both hands, with some fingers missing because of leprosy. He described that his hands and feet were left feeling numb and he also had difficulty in seeing from one eye. We were unable to identify anyone in his current surroundings to speak to despite description throughout the narrative that the relationship with others in the household was fine now. The illness began in 1979 where he sought treatment at the hospital and was told repeatedly that he had malaria/filaria and was treated accordingly. At this time, he was living in Monrovia and working for a large steel company. He had moved to Monrovia from Nimba county, where he had been living with a man known to the family who was supporting him through school. He moved to Monrovia to earn money. In 1999, during the conflict, he was in Ivory Coast, where they told him that he had leprosy. When he returned from Ivory Coast in 2000, he went straight to the leprosy treatment centre, where he was treated for Leprosy for 4 years and 10 months, returning to the community in 2004. Throughout the narrative he is very specific with dates of events.

**Case Study Summary CS011**

Female, over 49, living with mobility restrictions in her left leg and vision problems in her left eye as a result of leprosy. She was a widow and described the death of husband before she got sick. The participants sister was the other individual included in this case study who she now lived with and appeared to have a close relationship with following the death of her husband. She had been treated as an in-patient for Leprosy in 2003, having been referred from the clinic in her community. At the point of treatment seeking she had been living with symptoms of leprosy for 10 years. She described a happy childhood with a small family, she had married young when her father accepted a proposal for her. Once married, her and her sister described her as a breadwinner within the family, particularly because she could *‘hunt like a man’.*  Leprosy had impacted both her and her sisters’ life quite dramatically, particularly in terms of their economic status, now very reliant on handouts from the community, who they used to support, and who now don’t want to provide things for them for free. Throughout the narratives there were intersections with age in terms of social exclusion and interactions with the community.

**Case Study Summary CS012 and Case Study Summary CS018 (these participants were married and so both were interviewed as well as CS012’s daughter)**

Male, over 49, experience of gnarling in both hands and problems with his feet as a result of Leprosy. His wife (person affected linked to CS018), is also living with the effects of Leprosy, specifically, having lost one foot and on crutches and also some gnarling in both hands. She is also over 49. Both are living in a peri-urban settlement just outside the leprosy treatment facility which was a previous leprosy colony. The couple had met following Leprosy treatment and married at this time. He found comfort in the fact that they had both experienced Leprosy stating that *‘you are sick, I am sick, when you and I are together, I will not feel hurt’* (CS012). We also spoke to his daughter to complete the case study and asked both participants about the experience of their spouse. The information that the participants daughter could provide us with was limited as she had been told for most of her life that her father had died as he had been forced out of the community following Leprosy treatment. She had only recently come to find her father but was able to share information about how she felt about the situation and the impact the ‘loss’ of her father had had on her. The interaction between the participants and the daughter provided detailed information about the shared experience of leprosy and what it was like living in Wuo Town. The male participant translated at times for his wife, he also sometimes added information to her story, but did not appear to restrict the experiences she shared, although may have limited any negative description of their interpersonal experience.

**Case Study Summary CS014- see Box Two- Hannah’s Story**

**Case Study Summary CS015**

Male, over 49, blind following onchocerciasis. When he first got sick it was the war time so he described spending 2 years trying to seek help at the country doctor before going to the hospital. He had to leave school due to the sickness. He was married and described that it is his wife who is looking after him now. He described living in a constant cycle of debt where they would pay one person back for something and then have to gain credit from someone else. Borrowing money was common to pay for their son’s school fees, food or medical expenses. His wife had previously been married to his brother, however when his brother died during the conflict they had married as this was customary. He was already blind at this point. The wife described finding it distressing to look after her husband and that people in the community would sometimes talk badly to her because of him being blind- she it was a curse or witchcraft. They described wanting a poultry farm to help reduce the amount of debt experienced.

**Case Study Summary CS016 –** Excluded non-NTD case study, only became apparent during narrative.

**Case Study Summary CS017**

Female, 25-49, treated as an outpatient for Buruli Ulcer and had no physical limitations remaining. Had been ill with leprosy as a child. This participant was living alone with her young daughter and could not identify anyone with whom we could also do a household interview. She was a fufu seller in the market and asked us to also visit her there for her follow up interview. When asked about her interactions with the community and her family she became very emotionally distressed describing that there was no one around her anymore as they had either died or left her alone. She was living in a one room dwelling and described doing all she could to support her daughter to go to school. She described struggling to stand as it caused her pain if she stood too long and so preferred sitting. She described the cause of her disease as being linked to something that happened to her on her cassava farm as when she returned she had a blister and burning feeling in her leg that meant she couldn’t sleep. Throughout the interaction she described changes in the community that had made her feel sad, she attributed changes to the war. For example, before the war when she received leprosy treatment this had all been free, however now when she went to access services she had to pay. She also described that everyone used to know and support each other within the community but that this had stopped since the war time.

**Case Study Summary CS018-** see summary CS012

**Case Study Summary CS019**

Male, 25-49, some gnarling in his hands, skin discoloration and problems with sight as a result of Leprosy. Currently being treated as an in-patient at leprosy treatment facility. Has never been to school and was engaged with farm work in community before he got sick. His symptoms began in 2016 where he sought treatment at the traditional healer as he thought the illness was due to ‘African signs’. He described seeking treatment at the country doctor as it was what his parents would do. This cost the family a significant amount of money in cash and in items such as chickens which led to him feeling bad. He described the need for treatment and feeling ill consuming all his daily activity. When treatment failed here, another member of the community told him to travel to the leprosy treatment facility where they would have treatment for what he was experiencing. The journey to the facility cost 20USD where he was now receiving in-patient treatment and focused on getting well. He had concerns about the impact of the disease on his ability to complete ‘hard work’ or farming when he returned to the community. He also described being reliant on his uncle to visit him for company and money as his father was sick and mother too far away

**Case Study Summary CS020**

Female, 18-25, sore on foot because of leprosy, been a patient at leprosy treatment facility for 2 years and 4 months. Prior to illness was living in a house with a woman who was violent towards her because she could not contribute to the household as much another girl who was given money by her boyfriend to contribute. The woman who owned the house suggested that she engage in ‘man business’ (sex work) to be able to contribute. She was involved with one man who got her pregnant and then left her, where she had to return to her grandmother’s house to give birth. She described having given birth in her grandmother’s house and that when her father had been told about the birth he was very angry, refused to believe it and threatened to kill her with a cutlass (large knife used for farming). Originally from Ivory Coast, travelled for treatment, found it difficult to express story properly in the languages available. Prior to coming to leprosy treatment facility, she described that people were already beating her and trying to force her from the community when she experienced symptoms. As a result, following a failed attempt to visit the facility and then the herbalist (when instructed by her uncle), she described leaving her grandmother’s home to travel to the leprosy treatment facility, she described a lot of fear around this as she had never been to Liberia before and she knew that she would lose contact with her family. This journey was made one week after giving birth. Since arriving in the facility, she has had no contact with her friends and family. Travelling to Ganta cost her 40USD, however she described liking it there because people were accepting of her and her children and they divide food and soap for them monthly: *‘people at [at the treatment facility] hold and support me’*. She described a lot of fear at the prospect of having to go back to her community as she knows she will be treated as she was before. She knows it is not good for her children to spend their whole life at the hospital however if she must leave the treatment facility she would rather find a *‘new place to go with them instead of going back home’*. When asked how she felt about the sickness at different point she described that ‘*I feel bad and think about committing suicide. Because I was not even allowed to enter the church. So, I used to go beside the church and pray with my hands on my kids’.* She described mixing water and rat poison at several points to try and kill herself but had stopped herself due to fear of what would happen to her children. She described that these suicidal thoughts had decreased since being at the treatment facility, however when she was sitting on her own, she can often begin to feel bad again when thinking about the past and future.

**Case Study Summary CS021**

Male, 18-25, lesions on face and arms as a result of leprosy, been a patient at leprosy treatment facility since 2016. Prior to illness was in school (eight grade), but when started getting sick, had to drop out of school. His parents were completing contract (farming etc) work to help support him in going to school. When he started experiencing symptoms, his mother took him to the country doctor. He didn’t feel this was necessary as he knew no one in his family would bewitch him and he was concerned that his mother was spending a lot of money for this treatment. The disease progressed and started to appear on his face, at this point, someone also came to the community and advised that he should go to the clinic. His mother took him to the clinic in Bong county where they indicated that he may have leprosy and should go for treatment at the treatment centre as this was the only place that it was available. When they returned to the community, his mother engaged in more contract work to be able to provide the money to take him to the facility. When they finally reached the facility and they told him that it was definitely leprosy, his mother left and hasn’t returned. He described struggling with this and often can’t afford to buy clothes and is reliant on someone providing these things for him. Before leaving his community, he described that people had already started to exclude him because he was sick. He described that friends stopped interacting with him and he was made to eat separately. He feared that when he was better he wouldn’t be able to return to the community as he would still be treated the same. He hopes that once he is better he can be supported to make a business so that he would be able to return to school.

**Case Study Summary CS022**

Female, aged 26-49, partnered although described being left by partner to look after the children just before the sickness in 2009. She described been taken from school to be with this man at a young age as ‘people were blind to education then’. Since she has got sick, he has not cared for her or her children. Currently a patient at leprosy treatment facility since 2016 and has challenges with her feet because of leprosy. She described the illness beginning in 2012 when she was 39 years old and a long period of four years where she made repeated visits to different health care providers to seek diagnosis and treatment. She described that this period of health seeking had cost approximately 35USD and that she had had to rely on finances and support from family and the community during this time despite trying to also complete her normal farming and selling work. During this period when the sickness was at its worst she described being unable to complete basic household tasks such as dressing and drawing water. She first sought treatment at the traditional healer who tried to use chalk to heal her, when this failed she described travelling to Monrovia and other large centres in search of treatment. She also sought a lot of advice and searched for help in various churches and she believed that god led her to the treatment facility in a dream. When she finally received a diagnosis at the treatment facility she described feeling relieved to know what was wrong with her. During her treatment at the leprosy treatment centre she described several episodes of chronic pain where she had to seek additional treatments. She also described calling her son to talk to him when she was experiencing this pain and then he tries to come and visit her at the weekends. Her other children are too young and live too far to visit her which she found particularly distressing. Prior to getting sick, she engaged in several small business activities between Liberia and Ivory Coast and she used this money to prioritise her children’s education. Since getting sick, she described the main impact on her and her children was that they were no longer able to go back to school although she is trying her best to support them. She feared getting better and the challenge that this will bring of trying to start a new life for her and her children as she didn’t feel like the community would accept her if she was to return.

**Bong Case Studies**

**Case Study Summary CS023**

Male, 25-49, currently an inpatient because of Buruli Ulcer. He is a cassava farmer and described returning from the swamp and experiencing sores on the skin. He went to the country doctor immediately within one week of getting sick who gave him some cream to rub on the sore. This didn’t work, and the sore got bigger and burst, he became very sick and was shivering and vomiting and thought that he was going to die. A group in the community that he was part of put money together to try to help him and a friend told him that he should go to a specific treatment centre for the foot business and so that’s when he travelled (about a year after the symptoms). He described that he went to the country doctor first as they are ‘uncivilised people’ who want to deal with the witching first. He said that although no one has been to visit him since he arrived at the facility, people have been sending him money on a motorbike to help him. He feels like when he is discharged he will be able to return to the community and he will be ok and will be happy. He described that he will try to go to the swamp less and would like to spend time selling as oppose to being on the farm. His main worry was that when he got sick he had had to sell everything to pay for treatment and he is not sure how he will look after his wife and send his children to school, especially if he is no longer able to do any hard work.

**Case Study Summary CS024**

Female, age 18, Buruli Ulcer patient. The ulcer was on her right leg and has left severe scaring, when she was talking to us she kept this hidden the whole time and used her left leg to describe what had been happening to her. She was living at home with her family and selling coal in the community to survive having left school in 8^th^ grade as her family could no longer afford her school fees. She woke up one morning and her foot was puffy and swollen so her mother carried her to her uncle who made traditional medicines for her to treat her foot as they thought that it was caused by African signs. After one year when the sore did not improve, her mother carried her to her aunties house so that she could go to the facility for treatment. Her mother spent some time with her there before returning to the community. During the treatment she had an operation on her foot which cost 50USD as well as travelling to the clinic three times per week for injections and to manage her wounds. She described that the wound on her leg had healed, however she now has sore on the back of her hand. She said that she now found it hard to move about now because of the sore; she can’t draw water; she can’t complete household tasks and her sister must help her. Since her sickness she felt that the same friends tend not to come around her anymore which can make her feel down and even think about committing suicide but she doesn’t have anyone to talk to about feeling bad like that. She described that this had improved a little since the healing of the wound on her left and that some of her friends come around her a little more again now, which has helped to reduce her feeling suicidal. During the sickness, she also described that her family were treating her bad, she was reluctant to talk about it and didn’t want to tell us how they had been treating her. She described that she was not able to talk to anyone about it. In the future she hopes to return to school so that she can progress to further education to be able to provide for her family.

**Case Study Summary CS025**

Male, 44 years old, has large ulceration due to Buruli Ulcer on left leg. Left Bong county in 2011 to move to Monrovia where he was working as a construction worker before the illness started. The sore started in January (two years ago) with a pain in his waist. When he experienced the pain, he talked to a friend and his brother and sister about it. They told him it was African signs and so he went in the ‘sick bush’. He spent 8 months in the sick bush, but the ‘medicine man’ told him to go to the hospital after the wound burst 6 times. He spent about 175 USD in the sick bush. His brother described calling him to come back to Bong county for treatment where the family would be able to support him. He made the decision to return because ‘my parents are here. In Monrovia it is very expensive, so I decided to come up here to get well to go back to my family’. His brother described that he is currently supporting the whole household with some contract work. In October, he went to phoebe for treatment where he had an x-ray and then was given tablets which he returns for more of when they are finished. He has been told to be patient and to wait for the NTD team to come and to see the bone doctor. There was some level of frustration as he had been waiting for this for a long time and wanted us to provide treatment for him. The need for a ‘bone doctor’ had caused him some confusion as he doesn’t believe that anything has happened to his bone; ‘I didn’t experience any trauma for it to be my bone that is sore’. He described believing that the cause of his sickness is witch craft and referred to the ability of god to take it away from him. He described improving having not been able to walk for four months but slowly he can stand again thanks to god. He was however frustrated the he had now been sick for over two years. He described feeling very bad as he had had to leave his wife and children behind in Monrovia. He felt that ‘because I am the man I supposed to be that thing’ and worries most about his children and who will take care of them. During periods when he is feeling very bad, because his ‘movement is limited, activities in life ends’ can feel so bad that thinks about committing suicide. He described that some of his friend’s sill come around him and talk to him and this can help him to feel better. Although his brother described that it is only friends that know him very well who will still interact with him. Some youth group members (of which he was part before his illness), got together to send him cash to support with buying food etc.

**Case Study Summary CS026**

Male, age 26-49, Buruli Ulcer out-patient-Phebe hospital, Bong county. He described having a relatively good life before the sickness and that someone from America had paid for him to go to school until the conflict when he stopped because there were no funds anymore. Since this time, he was selling things in Monrovia until the sore started when he had to return to Bong county as treatment is cheaper there. He is currently selling books and he uses whatever money he makes each day to eat. Before getting sick he was living with a woman, however because of the sore his family won’t let him sleep there anymore. He decided to leave the house for himself because ‘if he used the same bucket they will be talking’. He described that his wife left him ‘she can’t be suffering under this man’ I can’t provide for her. We also spoke to his niece as someone who was important to him, they were clearly very close, and he had provided a lot for her schooling. They were currently living separately as the house where most of the family live only had one room, he was staying in a place nearby. Most of their other family live outside of Liberia, they left during the war and they don’t have contact with them or they don’t want to help him. His niece described that she wants to know more about what is wrong with him to help him as he is the only person who has been helping her with his schooling.

**Case Study Summary CS027**

Female, over 49, visual challenges and skin condition because of onchocerciasis. She was a widow currently living with her children in Bong county. She described being married and her husband having four wives who all used to cook and eat together. She described going to work everyday on the plantations when she was younger as well as being part of the church women’s group. She also reflected a lot about the only other time she had experienced illness was when she had measles as a child. Her son described the eye problem starting on his mother in 2012, however she had experienced itching before this time. The community only got a clinic in 2014, so they described being told by someone in the community that it was filaria and they should take her to the main referral hospital for the medicines. At this time, they didn’t have the money to travel to Phebe. When they had enough money, the trip cost them 40USD. On arrival at the county referral hospital they were prescribed the medicines and told to go and buy them from the pharmacy, but they were unavailable. They contacted family in Monrovia to try and help in obtaining the medicines, but this was not possible. As well as ‘formal’ health seeking, she also described making trips to the country doctor who would give her ointment for her eyes. She described that he told her she was going to go blind because of ‘sinking fontanelle’ which she said she took seriously as ‘if you don’t then something bad can happen to you’. Her son described being educated and so not believing in the need for country medicine, however it was still important to go to the country doctor because his mother and uncle said it was critical.

She described living with her daughter who is feeding her as she unable to do any farm work anymore. This meant that she is having to depend on god now because she can’t do anything anymore. Her daughter tells her that it is her duty to look after her and this was confirmed by her son who felt it was there responsibility to look after her despite the economic burden it was having on the household. Despite receiving care from her family, she described that this can make her to feel bad because all of her friends can make pepper farm, but she isn’t able and now she is just sitting with her children. She described that she wasn’t sure whether this was because of her illness or old age, but that she just must pray to god that she won’t go blind completely. Because of this, she was keen to take all the medicines that she was given and comes to the facility anytime she feels sick. She had told the facility staff a lot about the eye problems but less about the bumps on her skin. She described being told a lot that it was filaria after she has been given the medicine, but that she didn’t know a lot about the disease itself. Her son described that some people in the community can mock at her and she talks to him about it making her feel bad. She interacts with the family fine ‘only the bathing things we don’t share with her’, but that they try to keep the condition a secret from others because ‘that’s what is done in the interior’.

**Case Study Summary CS028-** see Box One Jon’s Story
